# Macrophages: A communication network linking *Porphyromonas gingivalis* infection and associated systemic diseases

**DOI:** 10.3389/fimmu.2022.952040

**Published:** 2022-07-27

**Authors:** Jie Lin, Dingming Huang, Hongwei Xu, Fenghuang Zhan, XueLian Tan

**Affiliations:** ^1^ State Key Laboratory of Oral Diseases and National Clinical Research Center for Oral Diseases and Department of Endodontics, West China Hospital of Stomatology, Sichuan University, Chengdu, China; ^2^ Myeloma Center, Winthrop P. Rockefeller Cancer Institute, Department of Internal Medicine, University of Arkansas for Medical Sciences (UAMS), Little Rock, AR, United States; ^3^ Division of Hematology and Oncology, Department of Internal Medicine, University of Iowa, Iowa, IA, United States

**Keywords:** *Porphyromonas gingivalis*, macrophages, immune escape, systemic disease, polarization, inflammasome

## Abstract

*Porphyromonas gingivalis* (*P. gingivalis*) is a Gram-negative anaerobic pathogen that is involved in the pathogenesis of periodontitis and systemic diseases. *P. gingivalis* has recently been detected in rheumatoid arthritis (RA), cardiovascular disease, and tumors, as well as Alzheimer’s disease (AD), and the presence of *P. gingivalis* in these diseases are correlated with poor prognosis. Macrophages are major innate immune cells which modulate immune responses against pathogens, however, multiple bacteria have evolved abilities to evade or even subvert the macrophages’ immune response, in which subsequently promote the diseases’ initiation and progression. *P. gingivalis* as a keystone pathogen of periodontitis has received increasing attention for the onset and development of systemic diseases. *P. gingivalis* induces macrophage polarization and inflammasome activation. It also causes immune response evasion which plays important roles in promoting inflammatory diseases, autoimmune diseases, and tumor development. In this review, we summarize recent discoveries on the interaction of *P. gingivalis* and macrophages in relevant disease development and progression, such as periodontitis, atherosclerosis, RA, AD, and cancers, aiming to provide an in-depth mechanistic understanding of this interaction and potential therapeutic strategies.

## Introduction

The innate immune system is the first line of defense against pathogenic organisms. Macrophages are a critical component of this innate immune response and are integral for initiating and sustaining the adaptive immune response. Macrophages were first discovered in the late 19th century (1) and are known to shape the host immune response to bacterial infection through phagocytosis, antigen presentation, and cytokine release (2). Macrophages are highly plastic cells of the innate immune system and play central roles in immunity against microbes, despite all these, macrophages also contribute to a wide array of pathologies depending on specific microenvironments. In atherosclerosis, macrophages are major players that form foam cells and mediate plaque stability (3). Microglial activation (the resident macrophages in the central nervous system) is a salient feature of neuroinflammation that is prominent in almost all neurodegenerative diseases (4). Tumor-associated macrophages (TAMs) are one of tumor-infiltrating immune cells suppressing immune surveillance. TAMs participate in tumor angiogenesis by secreting pro-angiogenic factors, including tumor necrosis factor-alpha (TNF-α), Interleukin-1β(IL-1β), C-C motif chemokine ligand 2 (CCL-2), and matrix metalloproteases (MMPs) (5), which are essential for metastasis and development of late-stage cancers (6).

*P. gingivalis* is an oral colonizing pathogen that requires hemin and iron for its growth and virulence. It is a Gram-negative anaerobic pathogen, producing numerous virulence factors such as fimbriae, capsules, lipopolysaccharide (LPS), lipoteichoic acids, gingipains, and outer membrane vesicles (OMVs)for its survival in hosts. Although *P. gingivalis* comprises only 0.8% of total clones in active human periodontitis, it is capable of remodeling benign microbiota into dysbiotic ones and is a putative keystone pathogen in the progression of periodontitis (7). Although the role of *P. gingivalis* acting as an oral pathogen is well-known, the effects of *P. gingivalis* extend beyond the oral cavity. In Alzheimer’s disease (AD), *P. gingivalis* induced peripheral amyloid β protein (Aβ) influx, aggravating the progress of AD (8). In patients with clinical and subclinical rheumatoid arthritis (RA), the number of *P. gingivalis* was increased (9); transcriptome of human blood samples also show that 14 periodontitis-associated pathways were significantly expressed during RA pathogenesis in RA patients and human gingival tissues from periodontitis patients (9). Furthermore, *P. gingivalis* infection are related to the production of anticitrullinated protein antibody (ACPA) in RA (10). Patients diagnosed with esophageal squamous cell carcinoma have poorer prognosis when *P. gingivalis* is detected. Higher levels of *P. gingivalis* have been revealed in infiltrating tumoral tissues than in normal ones (11, 12). These observations strongly suggest that *P. gingivalis* may play critical roles in systemic diseases.

*P. gingivalis’* role in systemic diseases were discovered, but the mechanisms of how *P. gingivalis* participate in these processes are rarely reported. We reviewed recent studies about *P. gingivalis* associated diseases and proposed that macrophages may serve as a communication network linking *P. gingivalis* infection and systemic diseases. *P. gingivalis* can invade and survive in resident macrophages, *P. gingivalis*-infected macrophages were observed to injure distant organs by producing cytotoxic extracellular vesicles in animal study (13). *P. gingivalis* uses complex strategies to evade the major antimicrobial mechanisms of macrophages, including pyroptosis (14), uncoupled inflammation and immune response, and disrupting phagosome-lysosome maturation (15). In this review, we will discuss how *P. gingivalis* acts on macrophages, evading the antimicrobial mechanisms of macrophages, and influences the polarization of macrophages in different microenvironments. The role of activation of NOD-LRR and pyrin domain-containing protein 3 (NLRP3) inflammasomes in macrophages by *P. gingivalis* will also be discussed. *P. gingivalis* will promote the polarization of macrophages and inflammasome activation if it is not eliminated by the hosts’ immune response. Thus, the survival and mechanisms of *P. gingivalis* from the hosts’ immune response will need to be studied and addressed. We also addressed that macrophages are a double-edged sword in response to microbial infection, its phenotype changes are essential in the processes of these diseases. An in-depth understanding of *P. gingivalis*-macrophage interaction with novel mechanistic insights into the pathogenesis of *P. gingivalis* with associated systemic diseases may be important for designing therapeutic strategies.

## Macrophage polarization in *P. gingivalis*-associated diseases

Macrophage polarization is the process in which macrophages are activated at a certain point in space and time and form different macrophage subtypes according to their environments (16). There are two major macrophage phenotypes, classically activated M1 macrophages and alternatively activated M2 macrophages (17). Different macrophage phenotypes have distinct functions. M1 macrophages can be polarized by granulocyte-macrophage colony-stimulating factor (GM-CSF), LPS, IFN-γ, and toll-like receptor (TLR) ligands, while M2 macrophages are polarized by macrophage colony-stimulating factor (M-CSF), IL-4, IL-13, IL-10, and immune complexes (18). M1 macrophages upregulate the expression of costimulatory molecules such as CD86 on the cell surface (18). M2 macrophages exhibit anti-inflammatory properties and express high levels of CD206, arginase-1 (Arg-1), IL-10, and transforming growth factor-beta (TGF-β), which negatively regulate M1 macrophage activity and contribute to the wound healing process (16, 18, 19).

**Figure 1:**
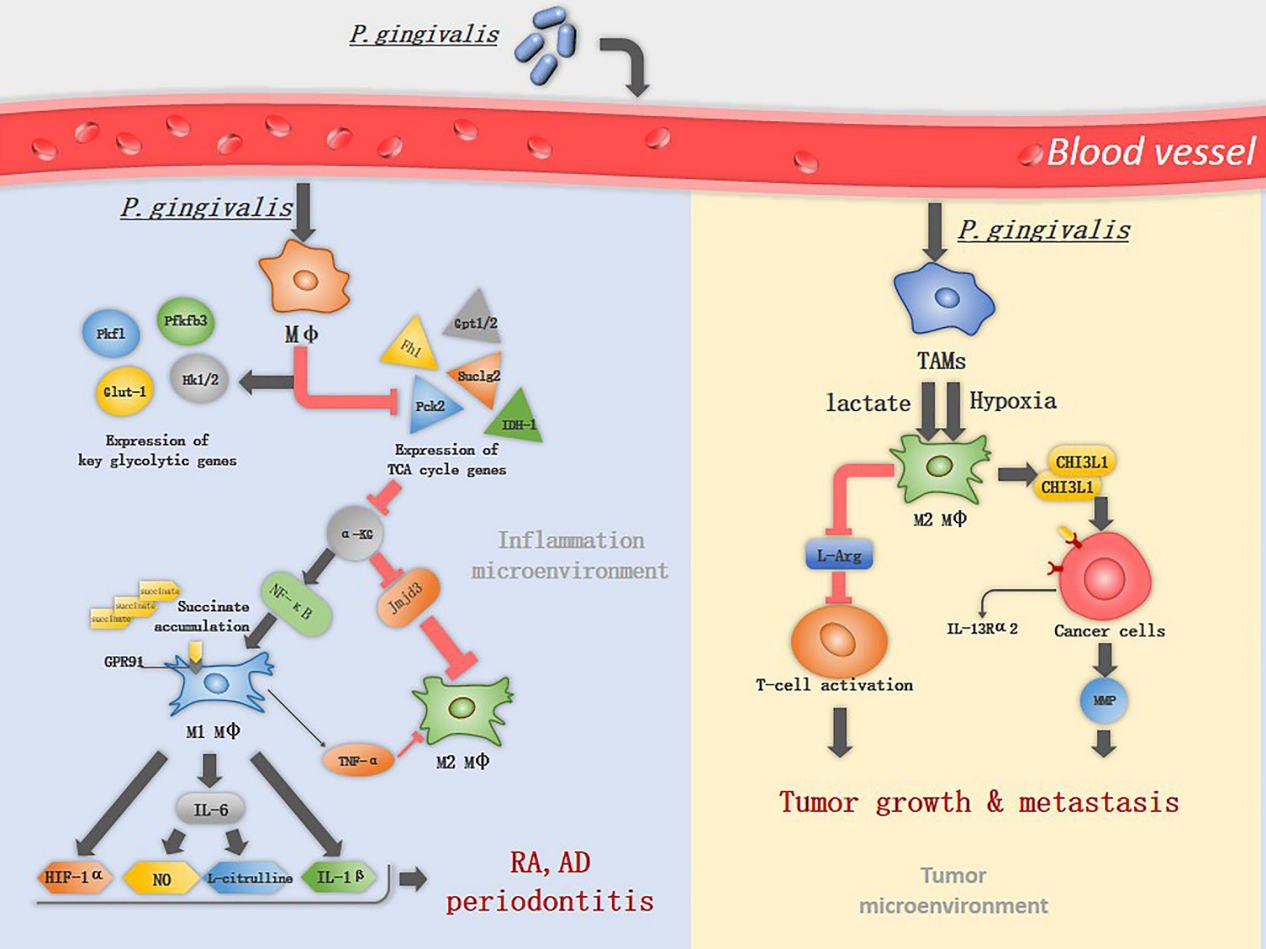
The mechanism of *P. gingivalis* in inducing macrophage polarization. *P. gingivalis* interacts with macrophages and increases the expression of key glycolytic genes, while inhibits the expression of TCA associated genes. α-KG is an important immunomodulator of M2 macrophage activation and can be suppressed by *P. gingivalis* through down-regulation of Idh1/2 and Gpt1/2, therefor induce classical M1 macrophage activation. M1 macrophages upregulate the expression of GPR91 and produce TNF-α, IL-1β, IL-6, NO, L-citrulline and HIF-1α, promoting the progress of RA and periodontitis. In TME, lactate and hypoxia promote TAM polarization into M2-like macrophages, which deplete L-Arg resulting in suppression of cytotoxic T-cell activation. M2 macrophages promote tumor metastasis by secreting CHI3L1 protein and MMPs, the CHI3L1 protein interacts with IL-13Rα2 expressing on the cancer cells, leading to upregulation of MMPs.

M1 macrophages have different metabolic profile compared to M2 macrophages. M1 macrophages utilize glycolysis metabolisms while in M2 macrophages the tricarboxylic acid (TCA) cycle has primacy over glycolysis (20). Metabolic reprogramming influences macrophage polarization (21, 22). In M1 macrophages the TCA cycle are broken in two places: after citrate and after succinate, which leads to citrate and succinate accumulation. Citrate was involved in producing NO, ROS, and prostaglandins (20). 24h after macrophages infected by *P. gingivalis* or its OMVs, will increase expression of glycolytic genes (e.g., *Glut-1*, *Hk1/2*, *Pfkfb*, and *Pkfl*) and will decrease TCA genes (e.g., *Fh1*, *Pck2*, and *Suclg2*) (23). *P. gingivalis* infection impairs TCA and suppresses α-KG production by down-regulation of *Idh1/2* and *Gpt1/2*, while it induces the accumulation of succinate (19). α-KG is a key metabolite that induces M2 macrophage polarization, prolylhydroxylases utilize α-KG as a substrate to destabilize HIF-1α.HIF-1α degradation suppresses glycolysis, decreasing M1 macrophage (24). Also, α-KG restricts M1 macrophage activation by downregulating the NF-κB pathway. It is a co-stimulator with Jmjd3. With their combination, they will regulate the trimethylation of histone H3, lysine 27 (H3K27me3) on promoter regions of genes that define the M2 phenotype, thus promoting M2 macrophage activation (25)(**Figure 1**). In addition, *P. gingivalis* activates TLR2 and TLR4 and elicits the expression of TNF-α in macrophages (26). TNF-α is an anti-M2 macrophage factor that blocks M2 macrophage polarization on two levels: through its direct effects on macrophages and the indirect effects of TNF-α on IL-13 production by other innate cell types (17).

An imbalanced M1/M2 ratio was discovered in various systemic diseases and tumors (27). Below we have listed well-studied examples such as periodontitis, RA, AD, and tumors, to reflect the interaction between *P. gingivalis* and macrophages.

### Periodontitis

Periodontitis is the sixth-most prevalent disease in the world, affecting many adults (28). Bacterial infection and immunological disorder are important elements of periodontitis. Gram-negative anaerobic bacteria that colonize subgingiva initiate the progression of periodontitis, and the host inflammatory response inflicts irreversible damage to the periodontal tissues (29). *P. gingivalis* is a putative keystone pathogen with the ability to impair innate immunity and transform a normally symbiotic microbiota into a dysbiotic state in periodontal tissue (7). Macrophages participate in the initiation of inflammation and are the major immune cells producing pro-inflammatory cytokines and mediating alveolar bone resorption in periodontitis (30). *P. gingivalis* induces M1macrophages by downregulating α-KG production (19), and M1 macrophages were the dominant phenotype for the gingival infiltration in response to *P. gingivalis* infection (31). Compared with healthy gingival tissues, the number of M1 macrophages and the M1/M2 ratio were enhanced (32). The production of pro-inflammatory cytokines characterizes M1 macrophages however, excessive or sustained pro-inflammatory cytokine production promotes periodontal destruction (33), and decreasing the M1/M2 ratio in mouse periodontitis models was proved effective in preventing alveolar bone loss (34). The newly developed nanocomposites which inhibit M1 macrophages’ polarization simultaneously initiate M2 macrophage polarization exhibiting a favorable repairment in periodontal soft tissue and decreased local periodontal inflammation in rat models, suggesting that the phenotypic switch of M1 to M2 might be a critical mechanism in mediating periodontal tissue repair.

### Rheumatoid arthritis

Rheumatoid arthritis (RA) is an autoimmune disease characterized by synovial inflammation and joint erosion, affecting 1–2% of the population worldwide (35). Research shows that oral microbiota dysbiosis is a high-risk factor for subclinical RA, *P. gingivalis* as a keystone pathogen inducing dysbiosis (9, 10), its DNA was found in the synovial tissue recently (36). In fact, peptidylarginine deiminase (PPAD) produced by *P. gingivalis* is able to citrullinate both endogenous and human proteins. Citrulline-specific autoimmunity is a key feature of RA, suggesting *P. gingivalis* may contribute to the development of RA (37). *P. gingivalis* exposure leads to anti-cyclic citrullinated peptide 2 (anti-CCP2) production, and subsequent bone resorption also confirms this hypothesis (38). Study has showed increased macrophages in the inflamed synovial membrane, and exhibit the potential of macrophages to be an early hallmark of active RA (39).Considering that macrophages are major cells responding to microorganism infection, it is very likely that the *P. gingivalis*-macrophages interaction is an important mechanism in RA.

Macrophages are predominantly the M1 phenotype in RA (16), which is known to produce many inflammatory cytokines that promote RA progression, including TNF-α, IL-1β, IL-12, IL-18, and IL-23 (40). Among these cytokines, TNF-α plays a major role and leads to the development of chronic polyarthritis, TNF-α and IL-6 together induce the differentiation of osteoclasts in a RANKL-independent manner (41, 42). *P. gingivalis* induces the M1 macrophage activation. The metabolic remodeling in M1 macrophages fuels the production of lactate and succinate, acidifying the extracellular space, which promotes the formation of a low-glucose and high-lactate microenvironments, a typical microenvironments in RA (43). Succinate accumulation leads to HIF-1α activation *via* inhibition of prolyl hydroxylases, HIF-1α stabilization and activation facilitate the metabolic shift from OXPHOS to glycolysis, therefore, sustaining the inflammatory phenotype of M1 macrophage (44). Extracellular accumulation of succinate activates the G protein-coupled receptor 91(GPR91) on macrophages, is involved in pro-angiogenesis of RA, facilitating immune cell extravasation into the synovium (45). iNOS is highly expressed in M1 macrophages, which is then catalyzed by L-Arg into NO and L-citrulline, L-citrulline is commonly accepted as a biomarker in RA (40) (**Figure 1**). Transforming M1 into M2 macrophages resulted in the reduction of clinical arthritis scores (40), suggesting that altered macrophages phenotype is a viable therapeutic option in RA.

### Alzheimer’s disease

Alzheimer’s disease (AD) is characterized by diminished cognitive function, specifically dysfunction of memory and judgment. The presence of extracellular Aβ marks pathologically AD. An anti-infectious agent in AD was proposed around 30 years ago on the basis of the discovery of herpes simplex virus 1 (HSV1) DNA in brain tissue in a high proportion of older people (46), and recently neuroinflammation has been suggested as a vital player in AD (47).

The role of *P. gingivalis* in neuroinflammation was discovered in recent studies, and small-molecule inhibitors targeting gingipains blocked *P.gingivalis*-induced neurodegeneration significantly (48, 49). Microglia are resident macrophages in the central nervous system constituting 5–10% of total brain cells, which changes their phenotype when stimulated by cytokines or LPS (50). The microglia-*P.gingivalis* interaction in the brain is related to the process of neuroinflammation. In rat brain, *P. gingivalis* LPS was discovered to activate microglia and increase the expression of TNF-α, IL-1β, IL-6, higher levels of the CD86 marker, and iNOS. INOS and CD86 are the landmarks of M1 microglia, were also found in *P. gingivalis* infected microglia (51, 52), illustrating that *P. gingivalis* LPS stimulates the M1 activation of microglia. It was observed in animal models that *P. gingivalis* trigger the polarization of M1 macrophages *via* TLR4/NF-κB signaling pathway (52). In peripheral circulation, macrophages are one of the pools for Aβ (53), *P. gingivalis* activates the NF-κB/cathepsin B pathway, promoting the generation of Aβ by macrophages (54), then peripheral Aβ is transferred into the brain by advanced glycation end products (RAGE) expressed on cerebral endothelial cells (8). *In vitro* cell experiment confirmed that Aβ could increase the expression of iNOS whereas it downregulated Arg-1 in microglia, inducing the M1 microglia (55).

Pro-inflammation cytokines released by M1 microglia can aggravate AD, leading to synaptic dysfunction, neuronal death and inhibition of neurogenesis (47). Microglia with M1 phenotype have decreased phagocytosis of Aβ, and extracellular Aβ is an important characteristic of AD (56). The shift of M1 microglia to M2 phenotype showed decreased expression of pro-inflammation cytokines, and an increased level of the triggering receptor expressed on myeloid cells (TREM2) on the surface of microglia, which plays an essential role in the clearance of Aβ (57). TREM2 overexpression induces microglial polarization towards the M2 phenotype by suppressing the NF-kB pathway, attenuates the cognitive impairment (50).

### Tumors

During early carcinogenesis, TAMs exhibit a higher degree of similarity to M1 macrophages. M1 macrophages exert anti-tumor functions, including directly mediating cytotoxicity and antibody-dependent cell-mediated cytotoxicity to kill tumor cells (58), therefore it can efficiently recognize and destroy cancer cells. However, in the later stages of carcinogenesis, various factors in tumor microenvironments (TME), such as low pH and hypoxia promote TAM polarization into M2-like macrophages (11). TAMs with numerous M2 macrophage characteristics are linked to poor prognosis in cancer (17). M2 macrophages produce growth factors, protease and vascular endothelial growth factors (59). They also suppress T-cell functions by depleting L-arginine (L-Arg) and L-tryptophan from the TME *via* the expression of arginase 1 (Arg1) and indoleamine 2,3-dioxygenase (IDO), respectively (6). L-Arg is an essential amino acid for the re-expression of T-cell receptors following antigen engagement on T cells (5). M2 macrophages promote tumor metastasis by secreting chitinase 3-like protein 1 (CHI3L1) protein. The CHI3L1 protein interacts with interleukin-13 receptor α2 chain (IL-13Rα2) on the cancer cells, leading to upregulation of MMPs (5) (**Figure 1**).

Increasing evidence support an association between *P. gingivalis* and colorectal carcinoma, oral and esophageal squamous cell cancer, pancreatic cancer (60–62). One report indicated that in TME, *P. gingivalis* could increase the M2/M1 ratio and upregulate the expression of genes encoding for protumor molecules in TAMs, but the specific mechanism is yet unclear (11). M1 macrophages utilize glycolysis to generate adenosine triphosphate (ATP), resulting in lactate accumulation. In TME, lactate is not only a metabolic by-product, it stimulates histone lactylation which increases the expression and genes associated with M2 macrophage polarization (63).Stimulation with LPS, 4h later, inflammatory response genes (for example, *Nos2*) were induced, while *Arg1* levels were markedly increased 24–48 h after M1 polarization. The late-phase switch to M2-like phenotype originated from increased histone lactylation (64). This may partly explain the function of *P. gingivalis* in TME.

## Inflammasome activation in macrophages induced by *P. gingivalis* is implicated in multiple diseases

Inflammasomes are large, multiprotein complexes localized in the cytoplasm of the cell that initiate proteolytic processing of pro-inflammatory cytokines pro-IL-1β and pro-IL-18 into mature inflammatory cytokines. Inflammasome activation plays a key role in innate immunity and is involved in some inflammatory diseases and tumors (3).

**Figure 2:**
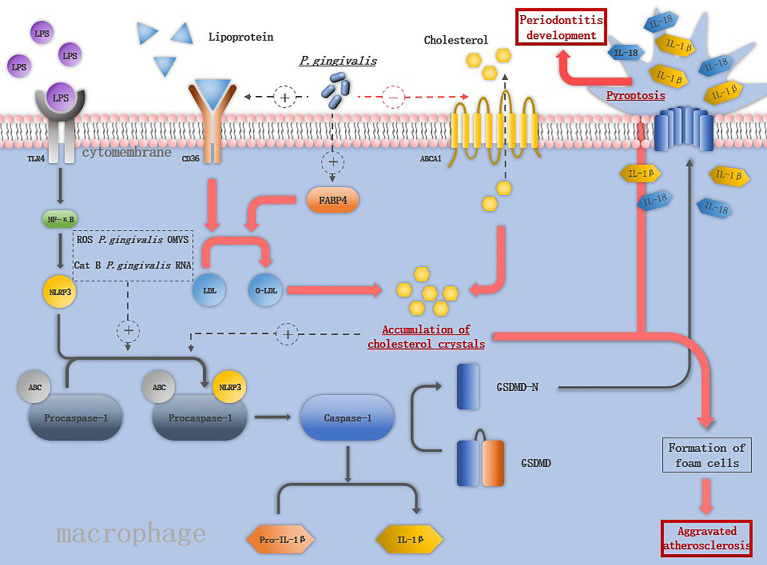
The mechanism of *P. gingivalis* in activating the inflammasomes in macrophage: LPS binds to TLR4 and activates NF-macrophage:increases the expression of keys are assembled in response to the second stimuli such as ROS, CatB, *P. gingivalis OMVS* and *P. gingivalis* RNA. The activation of NLRP3 inflammasome induces the maturation of IL-1ression of keys are assembled in response to the second stimuli such as ROS, Cs macrophage pyroptosis. Pore formation in the cell membrane leads to the release of IL-1β and IL-18 and promotes the destruction of periodontal tissue. *P. gingivalis* increases the expression of CD36 and FABP4, promoting the internalization of o-LDL and intracellular conversion of o-LDL to cholesterol crystals, which is able to activate NLRP3 inflammasomes. *P. gingivalis* also downregulates the ABCA1 on macrophages to promote cholesterol accumulation. The accumulation of cholesterol together with the releases of cellular components by pyroptosis contribute to the formation of foam cells, the hallmark cells of atherosclerosis.

Typical inflammasomes are constructed of pro-caspase-1, nucleotide-binding domain (NBD), and leucine-rich repeats (LRRs), called the NBD-LRR (NLR) superfamily that is responsible for the recognition of pathogen- associated molecular patterns (PAMPs) or other signals (65) and adapter molecule apoptosis-associated speck-like protein containing a caspase activation and recruitment domain (ASC) (**Figure 2**). Among these, caspase-1 cleaves pro-IL-1β and pro-IL-18, it also mediates their maturation and excretion (66). Inflammasome activation consists of two steps, an initial “cell priming” and a second “triggering” event, resulting in the proteolytic maturation and secretion of IL-1β (2).

### Periodontitis

Various cytokines produced by macrophages have long been believed to promote the development of periodontitis; inflammasome activation induces the maturation of two vital pro-inflammatory cytokines- IL-1β and IL-18 (67). Upregulation of inflammasome components caspase-1, NLRP3, and absent in melanoma 2 (AIM2) in gingival epithelial cells and macrophages of periodontitis patients suggests its role (68). NLRP3 inflammasomes is the most well-studied inflammasomes, experiments in mice models proved that alveolar bone loss induced by *P. gingivalis* infection was suppressed significantly in NLRP3^-KO^ mice (69). The activation of NLRP3 inflammasome needs two steps, firstly NF-κB signaling is activated and then NLRP3 inflammasomes are assembled in response to the second stimuli (70).

As important pro-inflammation cytokines source, macrophages interact with *P. gingivalis* activating NLRP3 inflammasomes. Compared to S. mitis, *P. gingivalis* promotes more robust secretion of IL-1β in macrophages by both NLRP3-caspase-1 canonical signaling pathway and NLRP3-caspase-4 signaling pathway (71). *P. gingivalis* induces the M1 macrophages accompanied by the release of HIF-1α, under hypoxia condition, augmented NLRP3 mRNA expression and more robust caspase-1 activation was detected in macrophages (72). Live *P. gingivalis* upregulates the miR-155 in macrophages to promote macrophage NLRP3 inflammasome activation (14). The virulence factors of *P. gingivalis* play different roles in inducing macrophage inflammasome activation. The OMVs of *P. gingivalis* included prime signal and the second signal. Both were needed to trigger the signaling cascade to activate macrophage inflammasomes and secretion of IL-1β (73), while gingipains apparently have a paradoxical role in activating inflammasomes, namely, to enhance caspase-1 activation and conversely, to cause proteolytic depletion of caspase-1 and IL-1β (23). NLRP3 inflammasome activation induce the macrophages pyroptosis, pyroptosis was initially described as a form of programmed cell death dependent on caspase-1, which was first described in 1992 in macrophages infected with *Shigella flexneri* (74). The prototypical form of pyroptosis is triggered by activation of pro-inflammatory caspases (caspase-1, -4, and -5 in humans and caspase-1 and -11 in mice) (75). Terminal cell lysis is then mediated through cleavage of gasdermin D (GSDMD) by one of these caspases (14, 76). Pore formation in the cell membrane leads to the release of IL-1β and IL-18 and promotes the destruction of periodontal tissue (76) (**Figure 2**). Pyroptosis may be an antimicrobial response of macrophages to eliminate intracellular *P. gingivalis*, it can also cause tissue injury, accelerate bacterial dissemination, and inhibit bacterial clearance from tissues (2).

### Atherosclerosis

Atherosclerosis, a complex multi-factorial chronic inflammatory disease, is characterized by the formation of atherosclerotic plaques. Macrophage numbers increase up to 20-fold within mouse aortas during atherogenesis (77), and the presence of cholesterol-engorged macrophage foam cells in atherosclerotic plaques is a hallmark of atherosclerosis (39). According to the newest hypotheses, inflammatory processes and lipid metabolism imbalance jointly contribute to the formation of atherosclerotic plaques in the arterial wall (78). *P. gingivalis* DNA was detected in atherosclerotic plaque in subjects with periodontitis. Clinical studies and animal models have reported that stimulation with *P. gingivalis* accelerates atherosclerosis (79, 80). *In vitro* research has shown that *P. gingivalis* can increase the expression of cell adhesion molecules, pro-inflammatory cytokines, and chemokines in endothelial cells, which have crucial roles in the recruitment of monocytes to the vascular endothelium and the subsequent formation of atherosclerotic plaques (81). The macrophages-*P. gingivalis* interaction in atherosclerosis is related to its initiation and progression.

A key feature of atherosclerosis is lipoprotein ingestion and accumulation by arterial macrophages *via* CD36. *P. gingivalis* increases the expression of CD36 and fatty acid binding protein 4 (FABP4) on macrophages, promoting the internalization of oxidized LDL (o-LDL) and intracellular conversion of o-LDL to cholesterol crystals (82, 83). Compared to nonatherosclerotic vessels, the mRNA level of NLRP3 inflammasome-related genes is significantly increased in human atherosclerotic plaques (84). NLRP3 inflammasome activation plays an important role in pathophysiology of atherosclerosis (**Figure 2**), and their silence was reported to cause the stabilization of atherosclerotic plaque (78). Cholesterol crystals in macrophages can damage the phagolysosomes and induce the release of cathepsin B, and along with *P. gingivalis*-induced ROS production in macrophages are able to activate NLRP3 inflammasomes. IL-1β is released through NLRP3 inflammasome activation and promotes the rupture of atherosclerotic plaques (70). Macrophages turn into foam cells in four steps: uptake of lipoproteins by CD36, hydrolysis of cholesterol esters, efflux of free cholesterol regulated by cholesterol transporters ATP-binding cassettes A1 and G1 (ABCA1 and ABCG1), and re-esterification of cytosolic cholesterol (85, 86). *P. gingivalis* downregulates the ABCA1 on macrophages and promotes cholesterol accumulation. IL-1β is a key cytokine in atherosclerosis, and IL-1β^-/-^ mice were reported to have a 30% reduction in the size of atherosclerotic plaques compared with the control group, in addition, monoclonal antibodies against IL-1β inhibit plaque formation in apo E^-/-^ mice (87). *P. gingivalis* activates the NLRP3 inflammasomes followed by macrophage pyroptosis, and macrophage pyroptosis releases cellular components into the plaque milieu, which is thought to contribute to the formation of the lipid-rich, acellular necrotic core, which characterizes vulnerable plaques (88).

## Macrophage immune response suppressed by *P. gingivalis* promotes disease progression

**Figure 3:**
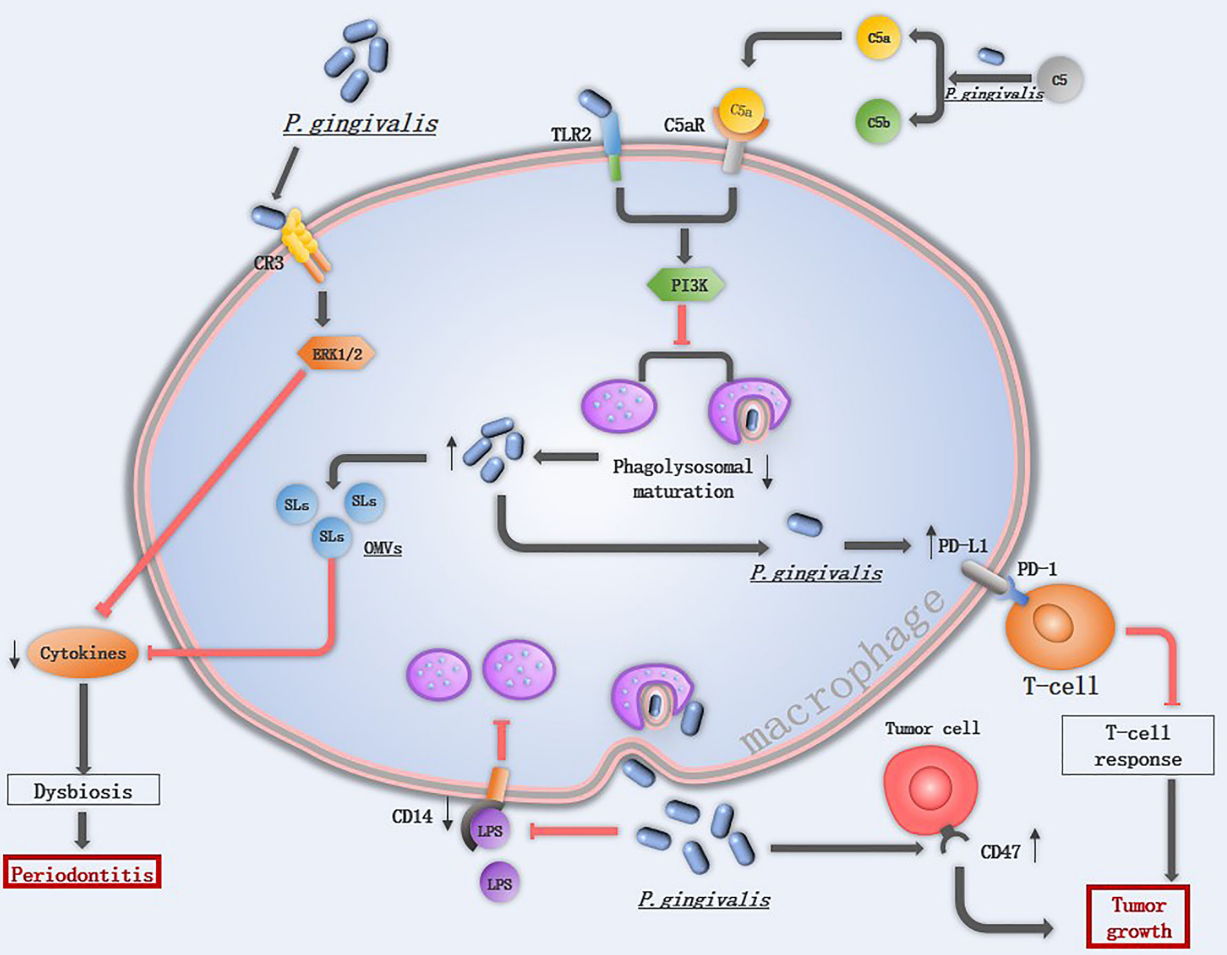
The mechanism of *P. gingivalis* in suppressing macrophage immune response: Extracellular P*. gingivalis* increases CR3 activation in macrophages while breakdowns C5 into C5a. The interaction of *P. gingivalis* with CR3 activates ERK 1/2, which reduces the cytokines level. The hemagglutinin/adhesion domain of gingipains cleave the LPS receptor CD14 from the surface of macrophages, resulted in a lower ability to phagocytize bacteria. The PD-L1 in macrophages is upregulated by *P. gingivalis*, which binds to PD-1 on T-cells and inhibits T-cell immune surveillance. CD47 is a phagocytosis inhibitor expressed on almost all cancer cells, it is regulated by *P. gingivalis* and protects cancer cells for macrophage phagocytosis. Once *P. gingivalis* enters into macrophages, intracellular *P. gingivalis* secretes OMVs containing SLs reduce the levels of pro-inflammatory cytokines, and initiates C5aR-TLR2 crosstalk signaling, activate the downstream PI3K pathway to suppress phagolysosomal maturation, avoiding being killed.

Survival of *P. gingivalis* from macrophages is an important prerequisite for *P. gingivalis* to start its promotion in the M1 macrophage polarization and activation of the inflammasomes*. P. gingivalis* can suppress macrophage immune responses by various mechanisms (**Figure 3**). Firstly, the capsule of *P. gingivalis* is able to reduce the ability of macrophage to phagocytose *P. gingivalis*, and the hemagglutinin/adhesion domain of gingipains can cleave the LPS receptor CD14 from the surface of macrophages, resulted in a lower ability to phagocytize bacteria (89, 90). When phagocytosed by macrophages, *P. gingivalis* is able to exit macrophages therefor avoiding being killed (91). Besides, *P. gingivalis* utilizes the complement C5a receptor 1-toll-like receptor 2 (C5aR1-TLR2) pathway to subvert immune response and suppresses phagolysosomal maturation, thus promotes intracellular survival (15). *P. gingivalis* releases sialidase which increases complement receptor 3 (CR3) activation in macrophages, then *P. gingivalis* interacts with CR3 and activates downstream extracellular signal-regulated kinases (ERK) 1/2, which reduces the level of IL-12p70 and inhibits IL-12-mediated clearance of pathogens (92). The inability of macrophages to clear *P. gingivalis* is associated with diverse diseases.

### Periodontitis

Toll-like receptors (TLRs) represent a conserved family of receptors involved in the detection of pathogen-associated molecular patterns (PAMPs) and the cellular response to bacterial invasion (89). TLR2 plays an important role in *P. gingivalis* infection. TLR2-deficient mice failed to induce alveolar bone resorption, and *P. gingivalis* cannot be detected in the gingival tissue of most Tlr2−/− mice at 24 h following oral challenge (15). Once TLR2 is activated, *P. gingivalis* initiates downstream phosphatidylinositol 3-kinase (PI3K) instead of myeloid differentiation factor 88 (MyD88)signaling pathway to escape immune clearance, TLR2- PI3K signaling suppressing the phagolysosomal maturation thereby escaping intracellular killing in macrophages (93). An *in vitro* experiment also demonstrated that *P. gingivalis* displayed a significant cycle of entering, exiting, and re-entering in macrophages to avoid being killed (91), implicating that low abundance of *P. gingivalis* may still induce severe tissue damage. Gingival tissues from healthy individuals and those with periodontitis contain sphingolipids (SLs), an amphipathic lipid that is essential for *P. gingivalis* to survive under oxidative stress. SLs can be transported to macrophages from *P. gingivalis* by OMVs and SLs reduce the levels of pro-inflammatory cytokines, therefore limiting the immune response to *P. gingivalis* (94).

### Tumors

*P. gingivalis* is able to promote cancer development by inhibiting apoptosis and accelerating gingival epithelial cell proliferation in the oral cavity. In addition to this, *P. gingivalis* also influences the immune response to oral cancer (11). One of the hallmark features of cancer is evasion of immune destruction. *P. gingivalis* and its LPS suppress the macrophage immune response to tumor cells mainly through upregulating PD-L1 in macrophages *via* the TLR-4 pathway (95). The peptidoglycan of *P. gingivalis* can induce PD-L1 expression on various cancer cells by a receptor-interacting protein kinase 2 (RIP2)-dependent mechanism or NOD1, NOD2 and MAPK dependent signaling pathways (96). Programmed cell death 1 (PD-1) is expressed on activated T cells, B cells, monocytes, and macrophages (97), the interaction of PD-L1 with its receptor PD-1 inhibits T-cell responses, and a blockade of this interaction has been proven to be an effective immunotherapy for several different cancers (98). CD47, a “don’t eat me” signal, is a phagocytosis inhibitor expressed on almost all cancer cells, and the ability of *P. gingivalis* to upregulate the expression level of *cd47* in cancer cells may be another mechanism to accelerate tumor development (11).

## Discussion

The innate immune system is the first line of defense against pathogenic organisms. Macrophages are a critical component of this innate immune response and are integral for initiating and sustaining the adaptive immune response. However, macrophages are a double-edged sword in the progression of host immune responses. In this review, we discussed the recently discovered mechanisms by which *P. gingivalis* induces macrophage polarization, activates the inflammasomes, evades the macrophage immune responses, showing that *P. gingivalis* induces the onset and progression of diseases not only by secreting various virulence factors but also by evading and subverting host innate immune responses.

However, there are still some limitations, in that the microenvironments surrounding macrophages is complex and they can be exposed to various cytokines, even those with opposite effects. The same molecular can play different roles in different microenvironments. For example, while HIF-1α is critical for M1 macrophage polarization, the same regulator participate in lactate-mediated M2 polarizations macrophage (24), hinting a role in both M1 and M2 macrophage polarizations, depending on the cues from the microenvironments. Due to the complex interplay of cytokines, metabolites and limited studies about the macrophages-*P gingivalis* interaction in tumor microenvironments, future research is expected to decipher it. Although the complexity of M1/M2 ratio, transforming the microglial polarization phenotype in mice models suggested reduced arthritis and learning and memory cognitive deficits (56, 99, 100) proved its therapeutic potentials. Considering the ability of *P. gingivalis* to induce macrophages M1 polarizations, blocking the macrophages-*P gingivalis* interaction may be a potential therapeutic method. In addition, although inflammasome activation is associated with various diseases and is discovered in *P. gingivalis-*infected macrophages, studies have also reported that *P. gingivalis* suppressed the inflammasome activation at the level of the second signal (101). This hint that the ability of *P. gingivalis* to activate inflammasomes is complex, and it’s necessary to uncover the function of various virulence factors in inflammasome activation. Besides, the divergent roles of NLRP3 inflammasomes are complex. It carries out both the pro-tumorigenic and anti-tumorigenic functions, host defenses against microorganisms such as *Candida albicans* and detrimental effects in other metabolic diseases including diabetes, obesity and atherosclerosis (102). However, the inhibition of IL-1β released by NLRP3 inflammasomes reduces cardiovascular burden in clinical data and mice studies, suggesting that modulating NLRP3 inflammasomes or the downstream cytokines is still a promising therapeutic direction in the future.

In this review, we summarized current studies about the macrophages phenotype changes infected by *P. gingivalis* and its systemic influence, although a more insightful mechanism of macrophages- *P. gingivalis* interaction is needed, and the mechanism of how *P. gingivalis* invades various tissues waiting to be determined, targeting macrophages in diverse diseases, reprogramming their phenotype and function, or clearing *P. gingivalis* and its virulence factors to block the macrophage-*P. gingivalis* interaction may be alternative therapeutic approach for inflammatory diseases, tumors, and autoimmune diseases. Considering *P. gingivalis* is an oral colonizing pathogen, sustaining a good oral hygiene or treating periodontitis may be beneficial to curing some systemic diseases.

## Author Contributions

JL, DH, HX, FZ, and XT wrote and revised the manuscript. All authors contributed to the article and approved the submitted version

## Funding

This work was supported by grants to XT from the National Natural Science Foundation of China (Grant No. 82001037) and the Research and Develop Program, West China Hospital of Stomatology, Sichuan University (Grant No. RD-02-202007), and grants to Dr. Tan from the Natural Science Foundation of Sichuan Province (Grant No. 2022NSFSC0751); grants to FZ from the National Cancer Institute (R01 CA236814) and US Department of Defense (DoD; CA180190), and funding from Myeloma Crowd Research Initiative Award (to FZ) and Riney Family Multiple Myeloma Research Program Fund (to FZ). And grants to DH from the National Natural Science Foundation of China (Grant No. 81970936).

## Conflict of Interest

The authors declare that the research was conducted in the absence of any commercial or financial relationships that could be construed as a potential conflict of interest.

## Publisher’s Note

All claims expressed in this article are solely those of the authors and do not necessarily represent those of their affiliated organizations, or those of the publisher, the editors and the reviewers. Any product that may be evaluated in this article, or claim that may be made by its manufacturer, is not guaranteed or endorsed by the publisher.
